# Prenatal Alcohol Exposure and Chorioamnionitis Results in Microstructural Brain Injury in a Preclinical Investigation

**Published:** 2020-02-17

**Authors:** Jessie R Maxwell, Tracylyn R Yellowhair, Suzy Davies, Danny A Rogers, Krystle L McCarson, Daniel D Savage, Lauren L Jantzie

**Affiliations:** 1 Department of Pediatrics, University of New Mexico, USA; 2 Department of Neurosciences, University of New Mexico, USA; 3 Department of Neurosurgery, Johns Hopkins University School of Medicine, USA; 4 Department of Pediatrics, University of Arkansas for Medical Sciences, USA; 5 Department of Pediatrics, Johns Hopkins University School of Medicine, USA; 6 Department of Neurology, Johns Hopkins University School of Medicine, USA; 7 Kennedy Krieger Institute, USA

**Keywords:** Chorioamnionitis, Prenatal alcohol exposure, Frontal cortex, Diffusion tensor imaging

## Abstract

**Background:**

Prenatal Alcohol Exposure (PAE) impacts 2% to 5% of infants born in the United States yearly. Women who consume alcohol during pregnancy have a five-fold increased rate of Chorioamnionitis (CHORIO). Both PAE and CHORIO cause microstructural injury to multiple brain regions including major white matter tracts.

**Objective:**

Utilizing two previously established animal models, we hypothesized that the combination of PAE+CHORIO would result in greater deficits in myelination and structural integrity than PAE alone.

**Material and Methods:**

Pregnant Long-Evans rats voluntarily drank 5% ethanol or saccharin until Gestational Day 19 (GD). On GD19, CHORIO was induced in one group of PAE dams by a 30 min uterine artery occlusion and injection of Lipopolysaccharide (LPS) into each amniotic sac. The remaining PAE dams and saccharin controls underwent sham surgery. Pups were born on GD22 and weaned on Postnatal Day 24 (PD). On PD28, offspring were sacrificed, and their brains examined using *ex-vivo* Diffusion Tensor Imaging (DTI).

**Results:**

Compared to control, PAE alone did not affect offspring birth weights, mortality or any DTI measures. In contrast, PAE+CHORIO significantly reduced offspring survival and, in surviving pups, increased Radial Diffusivity (RD) in medial frontal cortex and decreased Fractional Anisotropy (FA) in medial and ventral frontal cortex and within capsular regions.

**Conclusion:**

The combination of moderate PAE+CHORIO results in an increased mortality, concomitant with diffuse microstructural brain injury noted in young adolescent offspring at PD28. Future studies should examine the extent to which PAE exacerbates the damage caused by CHORIO alone and whether these deficits persist into adulthood.

## Introduction

Alcohol exposure in utero is associated with numerous life-long consequences including intellectual disability, impaired executive functioning, and poor motor control, including gait deficits [1]. Prenatal Alcohol Exposure (PAE) is a worldwide public health crisis with prevalence estimates exceeding 1% in 76 countries, but varying between countries [2,3]. South Africa has the highest prevalence of Fetal Alcohol Spectrum Disorder (FASD) at 111.1 per 1000, and at least 2% to 5% of infants in the United States are impacted by PAE [3,4]. Although the Centers for Disease Control and Prevention has recommended that women abstain from alcohol if they are pregnant or of child bearing age, as many as 1 in 10% women in the United States reported drinking alcohol on a monthly basis and 1 in 33% reported binge drinking in the last 30 days [5,6]. The consequences of alcohol use during pregnancy are widespread, and impact the fetus in multiple ways due to alcohol readily crossing the placenta and distributing into the fetal compartment [7]. Infants who develop Central Nervous System (CNS) problems, growth problems and specific abnormalities in facial features following in utero alcohol exposure are diagnosed with Fetal Alcohol Syndrome (FAS), which encompasses those most severely affected. However, many children with PAE do not develop the dysmorphic features included in the diagnosis of FAS. Therefore, the term Fetal Alcohol Spectrum Disorders (FASD) is more inclusive, and captures the whole range of physical, emotional and neurological effects present in children exposed to alcohol prenatally [8]. Across the spectrum of FASD, many children have impairments in cognition, self-regulation, and adaptive functioning [9]. The neurodevelopment of affected children with PAE evolves over time and involves abnormalities in global intellectual ability, difficulties with problem-solving and planning, poor inhibitory ability, poor adaptive skills, deficits in the ability to hold and manipulate information in working memory and impairment of both fine and gross motor skills [10,11]. These children suffer from specific life-long deficits in intellectual disability and attention problems that impact day to day function. Notably, although their Intelligence Quotient (IQ) may fall within normal limits, an individual with PAE can struggle with previously learned skills and have difficulties with nonverbal aspects of cognition [9]. Additionally, deficits in learning and memory for recently learned skills and areas of executive functioning are impacted in children with PAE, with higher rates of social behavioral problems and difficulty dealing with overstimulation when compared to individuals with attention deficit hyperactivity disorder [9]. The frontal cerebral cortex, one of the most complex brain regions with extensive interconnections, is keys to executive function and behavior including attention, planning and decision making; making this brain region critical for more detailed investigation in PAE offspring [12]. Given the global impacts PAE has on neurodevelopment, is apparent that the central nervous system is exquisitely sensitive to the effects of alcohol exposure during development and remains vulnerable throughout pregnancy [1,8,10,13]. Infants exposed to alcohol prenatally are also at a higher risk of poor neurological outcomes due to placental perfusion defects and inflammation, as observed in an infection of the placenta known as Chorioamnionitis (CHORIO) [14]. CHORIO, which is inflammation of the chorion, amnion and placenta, is one of the most common occurrences prior to spontaneous preterm birth [15]. Placental inflammation and infection are associated with an increased risk of spontaneous preterm delivery, thus increasing the risk of preterm delivery in women who drank alcohol during pregnancy [16]. Unfortunately, women who abuse alcohol during pregnancy have an increased risk of infections, including a 5 to 7 fold increase in rates of CHORIO [17]. Given the diffuse brain injuries that can occur separately with PAE or CHORIO [REFS], and the number of infants exposed to both alcohol and CHORIO during pregnancy, we hypothesized that CHORIO would exacerbate the damage induced by PAE, resulting in a unique pattern of changes on Magnetic Resonance Imaging (MRI), particularly in major white matter tracts, including the corpus callosum.

## Material and Methods

All experimental procedures were approved by the Institutional Animal Care and Use Committee of the University of New Mexico.

### Voluntary drinking paradigm

As previously described, pregnant Long-Evans rats (Harlan Industries, Indianapolis, IN) were single housed in plastic cages and maintained on a reverse 12 h dark, 12 h light schedule (lights off between 0900 and 2,100 h). Rat chow and tap water were available ad libitum [18,19]. The 20 dams used in this study were randomly assigned to one of three groups: 1. Saccharin + Sham Surgery (Control), 2. PAE + Sham Surgery (PAE) and 3. PAE + CHORIO Surgery (PAE+CHORIO). Prior to mating, all female rats were gradually acclimated to voluntary drinking of 5% ethanol in 0.066% saccharin for 4 h each day (1,000 h to 1,400 h). Following 2 weeks of 5% ethanol consumption, females drinking within 1 standard deviation of the group mean were paired with a proven male breeder until pregnancy was confirmed *via* the presence of a vaginal plug. Dams did not consume ethanol during breeding. Pregnant rat dams were then returned to single housing and voluntarily consumed either 5% ethanol or 0% ethanol for 4 h daily until Gestational Day 18 (GD). The total volume consumed by the 0% ethanol group was matched to the mean volume consumed by the 5% ethanol group dams. Previously, we reported that mean peak maternal serum ethanol concentration was 60.8 mg/dL and that this pattern of voluntary ethanol consumption does not affect maternal weight gain during pregnancy, placental wet weight at term, pup birth weight, pup mortality or offspring growth curves [18].

### Chorioamnionitis (CHORIO)

We have previously shown in a preclinical model that Transient Systemic Hypoxia Ischemia (TSHI) resulting in placental insufficiency and intra-amniotic Lipopolysaccharide (LPS) injections result in placental changes consistent with CHORIO, as well as a fetal inflammatory response syndrome [20–23]. Therefore, to induce CHORIO, a laparotomy was performed as previously described [20,22,24] with slight modifications for Long-Evans rats. On GD19, dams assigned to the PAE+CHORIO group were anesthetized with isoflurane and an open laparotomy performed. Uterine arteries were isolated and occluded with aneurysm clips for 30 min, after which the clips were removed and 4 μg of Lipopolysaccharide (LPS 0111:B4, Sigma, St. Louis, MO) was injected into each amniotic sac [20,22,23]. The laparotomy was closed in layers with sutures and the dams recovered. Rat dams in the Control and PAE only groups were exposed to isoflurane anesthesia with a laparotomy and identical anesthesia time without occlusion of the uterine arteries or LPS injection. Pups were matured with their dams until weaning on PD24 and then group housed with litter mates based on sex until PD28.

### Diffusion tensor imaging (DTI)

*Ex vivo* DTI was performed consistent with our prior reports [25,26]. Specifically, at Postnatal Day 28 (PD), rats received pentobarbital injections (50 to 100 mg/kg), and once unresponsive, were perfused with Phosphate Buffered Solution (PBS) followed by 4% Paraformaldehyde (PFA). The brains were removed and post-fixed in 4% PFA for 1 week. Brains were embedded in 2% agarose containing 3 mm sodium azide for *ex vivo* DTI. Scanning was completed on a Bruker 4.7-Tesla Bio Spec 47/40 ultra-shielded refrigerated nuclear magnetic resonance imaging system (Billerica, MA) equipped with a quadrature RF coil (72 mm i.d.) and a small-bore (12 cm i.d.) gradient set with a maximum gradient strength of 50 Gauss/cm. T2 and echo planar Diffusion Tensor Images (DTI) obtained in 8 to 10 continuous coronal 1 mm slices with a 2.00 cm field of view. T2 images had a 3000 ms TR and a 12 ms TE. DTI sequences were a 3000 ms TR, 40 ms TE and a 2000 mm^2^/s b-value with 30 Diffusion gradient directions. Bruker’s Paravision 5.1 imaging software was used to analyze all images by two observers blinded to prenatal exposure and surgical status. Regions of Interest (ROI) included corpus callosum, capsular white matter and the frontal cortex divided into medial and ventral regions. Directional Diffusion-weighted images and Fractional Anisotropy (FA) maps were generated. Fractional anisotropy, mean diffusivity (MD, [λ1+λ2+λ3]/3), axial diffusivity (AD, λ1) and radial diffusivity (RD, [λ2+λ3]/2) were analyzed and calculated. Each brain had 4 to 6 slices in which the corpus callosum and capsular white matter could be analyzed, with the values averaged per brain.

### Statistical analysis

The difference in ethanol consumption between the PAE and PAE+CHORIO groups was analyzed using a student’s two-tailed t-test. Differences in all other measures were assessed using a oneway ANOVA with Tukey’s correction between the groups. Data are presented as mean ± SEM with p<0.05 considered significant.

## Results

### Moderate prenatal alcohol exposure (PAE) and Chorioamnionitis (CHORIO) paradigm data

The impact of the moderate PAE paradigm alone as well as coupled with the CHORIO paradigm compared to the control group. There was no significant difference in the mean daily 4 h consumption of ethanol between the PAE and the PAE+CHORIO group dams. In a previous study, this level of ethanol consumption was shown to produce a mean peak maternal serum ethanol concentration of 60.8+5.8 mg/dL at 45 min after the introduction of the drinking tubes during the third week of gestation [19]. Maternal weight gain to Gestational Day 18 (GD) in the PAE alone and the PAE+CHORIO groups was not different compared to the control group. PAE alone did not affect litter size compared to the control group, and previously we have not observed any difference in pup birth weight following moderate PAE [19]. Further, there was no difference in mortality between the control and PAE groups. In contrast, the combination of PAE+CHORIO resulted in significantly reduced postnatal survival compared to the saccharin control or PAE alone groups. Specifically, the PAE+CHORIO combination resulted in 95% fetal mortality and death of live born offspring by Postnatal Day 2 (PD), with 92% of mortality occurring in utero.

### *Ex vivo* diffusion tensor imaging (DTI) analysis

**Figure 1:**
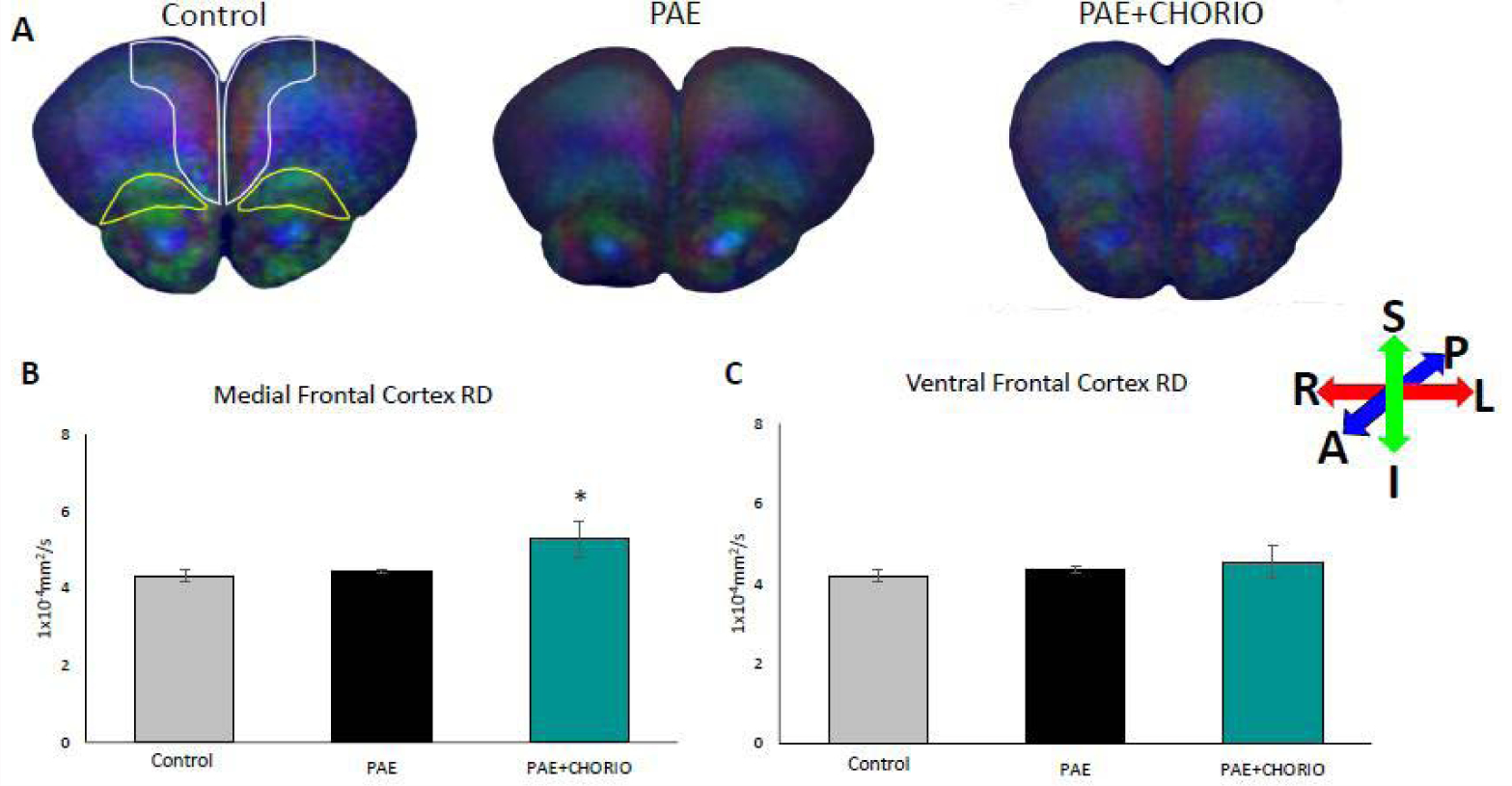
Prenatal alcohol exposure and chorioamnionitis results in microstructural injury in the frontal cortex. Directionally encoded color maps of diffusion of control, Prenatal Alcohol Exposure (PAE), and PAE plus Chorioamnionitis (CHORIO) brains at postnatal day (P) 28 are shown in A. Differences are noted in the frontal cortex brain regions, consistent with loss of microstructural integrity. The colored bars show diffusion along specific directions, with red representing left-right, green representing superior-inferior direction, and the blue coloration representing anterior-posterior direction. There is a significantly increasing Radial Diffusivity (RD) in PAE+CHORIO compared to PAE and control in the medial frontal cortex (B), that is not significant in the ventral frontal cortex (C). The regions of interest are highlighted in the control in A (white outline is the medial frontal cortex and yellow outline is the ventral frontal cortex). *p<0.05, n=3–5/group.

Figure 1 illustrates representative DTI color map images from each experimental group along with a summary of the radial diffusivity data collected on PD28 in two brain regions of interest, namely, the medial frontal cortex and the ventral frontal cortex. The directionally encoded color maps show water molecule Diffusion in X, Y and Z axes, where red indicates flow along the horizontal plane, green indicates flow along the vertical plane, and blue indicates flow along the orthogonal plane. The control image in Figure 1A denotes the areas where measurements were made in the medial frontal (white outlines) and ventral frontal (yellow outlines) cortices. Figure 1A illustrates directional Diffusion differences between the control, PAE and PAE+CHORIO brains, including attenuated Diffusion in the PAE+CHORIO frontal cortices compared to PAE and control, consistent with decreased microstructural organization of the tissue. Axial Diffusivity (AD), and mean diffusivity (MD) in the medial and ventral frontal cortices were not different among the three experimental groups (data not shown). However, there was a significant increase in the Radial Diffusion (RD) within the medial frontal cortex of the PAE+CHORIO compared to controls and PAE alone, as shown in Figure 1B. The ventral region of the frontal cortex had a trending increase in the RD values following PAE+CHORIO compared to controls and PAE alone, however this did not reach statistical significance (Figure 1C).

### Fractional anisotropy (FA) analysis

**Figure 2:**
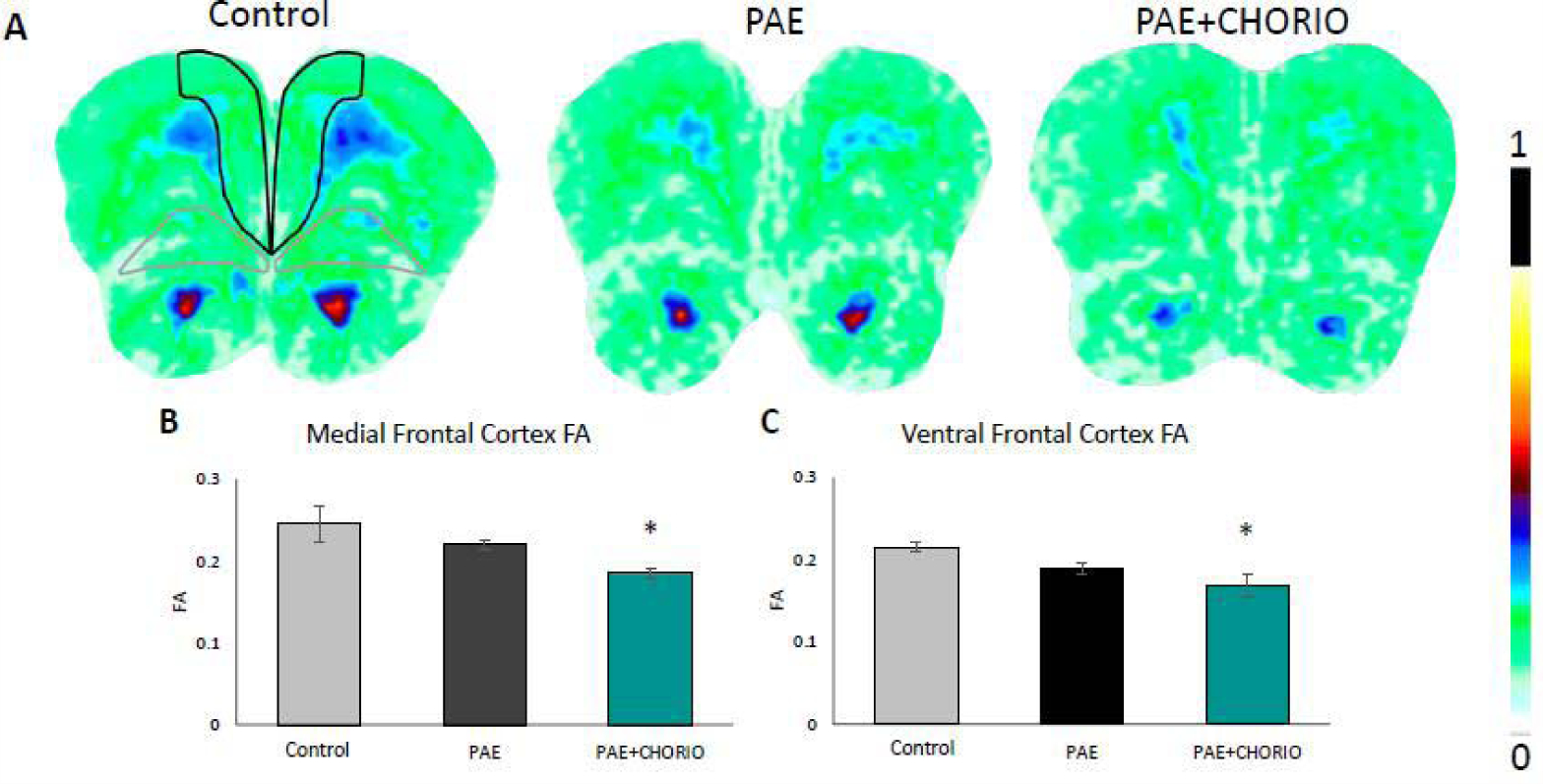
Fractional anisotropy in the frontal cortex is impaired following prenatal alcohol exposure and chorioamnionitis. Injury results in a significantly decreasing Fractional Anisotropy (FA) at postnatal day (P) 28 in two separate regions of the frontal cortex, the medial and ventral regions, (B,C) in Prenatal Alcohol Exposure (PAE) plus Chorioamnionitis (CHORIO) compared to control and PAE alone, as observed in the FA color maps. The colored bar to the right indicates that cool colors have a FA values closer to 0, while warm colors have a FA value closer to 1. Figure 2A shows the medial frontal cortex region of interest outlined in black, with the ventral frontal cortex outlined in gray. *p<0.05, n=3–5/group.

**Figure 3:**
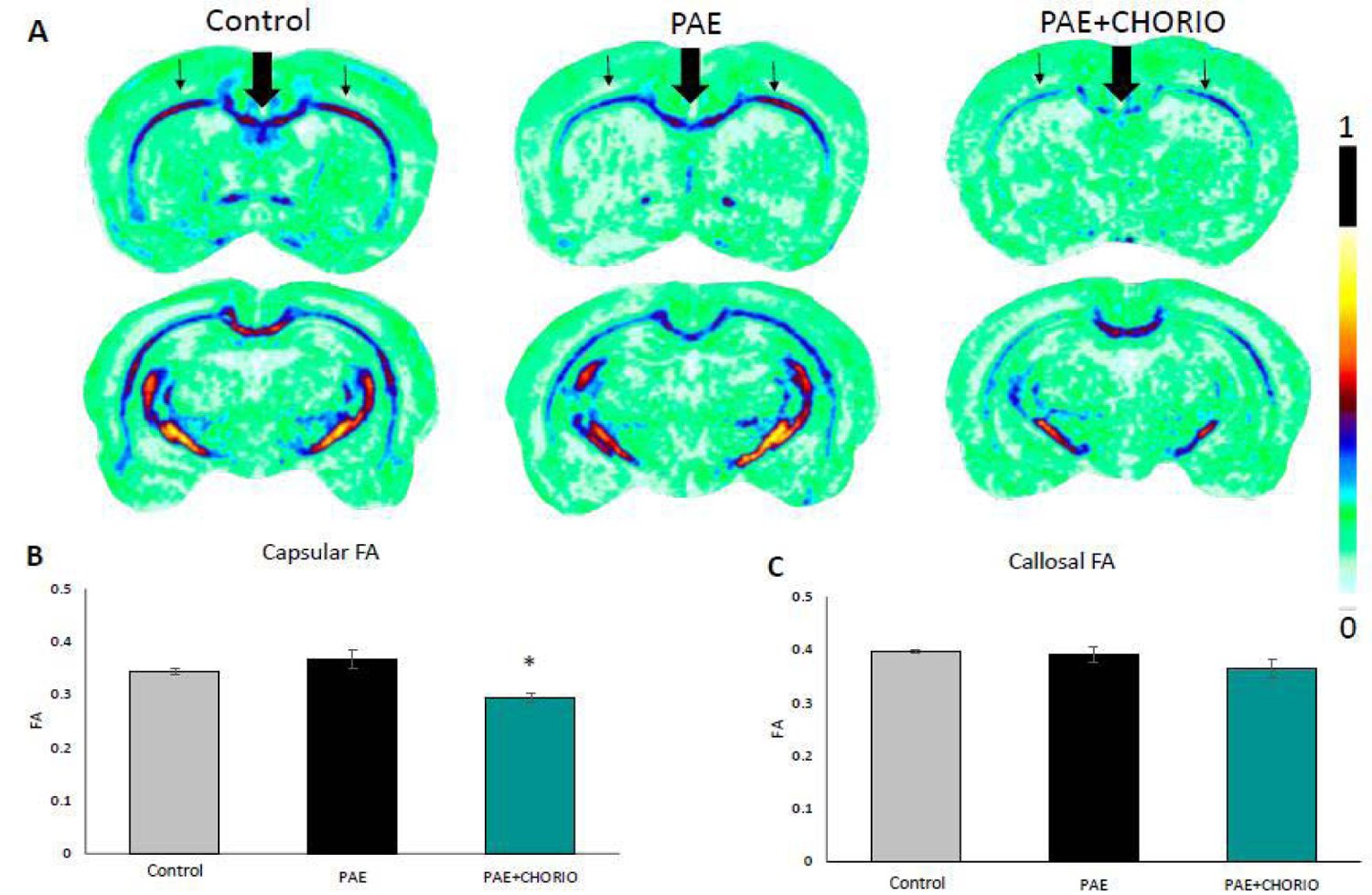
Microstructural brain injury in major white matter tracts following prenatal alcohol exposure and chorioamnionitis. Representative anterior and posterior slices of Fractional Anisotropy (FA) color maps of control, Prenatal Alcohol Exposure (PAE), and PAE plus Chorioamnionitis (CHORIO) brains at postnatal day (P) 28 are shown in A. The colored bar to the right indicates that cool colors have a FA values closer to 0, while warm colors have a FA value closer to 1. There are abnormalities in multiple areas, consistent with loss of microstructural integrity and coherence in major white matter tracts, specifically in the capsular white matter (thin arrow) and the corpus callosum (thick arrow). The FA in PAE+CHORIO was significantly decreased compared to PAE and control within the capsular white matter (B). Additionally, the corpus callosum had a trending decrease in FA that did not reach significance (C). *p<0.05, n=3–5/group.

Figure 2A displays representative color coded maps of Fractional Anisotropy (FA) in the three prenatal groups. FA is the degree of anisotropy, in which zero is isotropic movement, or unrestricted movement in all directions, and a value of one is anisotropic movement, in which Diffusion occurs along one axis only. The cooler colors represent FA values closer to 0 and warm colors represent FA values color to 1. Figure 2A shows the regions of interest outlined in the control image, in which the black outline represents the medial frontal cortex and the gray outline represents the ventral frontal cortex. The pattern of blue and green coloration in the control images in Figure 2A is consistent when compared to PAE, in which the blue coloration is less dominant. Compared to the control, there was a downward trend in FA in both the medial frontal and ventral frontal regions within the PAE group, however, this reduction was not significant. In contrast, there was a significant decrease in FA in both the medial frontal (Figure 2B) and ventral frontal cortices (Figure 2C) in the PAE+CHORIO compared to both the PAE and controls. In addition to FA reductions in gray matter regions of the frontal cortex, major white matter tracts displayed reduced FA in the brains for PAE+CHORIO offspring compared to the other two groups. Figure 3A shows color coded FA maps, where the thick arrow points to the corpus callosum and the thinner bilateral arrows point to capsular white matter. Again, FA was not different in either the corpus callosum or capsular regions of PAE brains compared to controls. In contrast, there was a significant decrease in capsular FA in the PAE+CHORIO group compared to both controls and PAE offspring (Figure 3B). While trending downward, the FA values in the callosal white matter in PAE+CHORIO did not reach significance when compared to controls and PAE (Figure 3C).

## Discussion

This is the first report of an animal model combining moderate prenatal alcohol exposure and chorioamnionitis, which resulted insignificant in utero injury and fetal demise. As previously published, the CHORIO model alone results in a mortality of 42% on average [22]. These data show that in the setting of placental insufficiency and infection, PAE results insignificantly increased late gestation fetal death, which is the clinical surrogate of spontaneous abortions in humans. Additionally, DTI analyses revealed a significant increase in radial Diffusion within the medial frontal cortex in PAE+CHORIO compared to controls or PAE alone. Finally, FA was significantly decreased within the medial and ventral frontal cortices as well as in the capsular white matter, confirming microstructural changes and decreased structural coherence of cerebral tissue. While we were not able to include a CHORIO alone group in the current study, it is noteworthy that our prior work assessing the impact of CHORIO alone on neurodevelopment and DTI changes, suggests that the combination of PAE+CHORIO here caused greater mortality than either PAE or CHORIO alone [27,28]. Consistent with our findings of increased mortality in PAE+CHORIO, consumption of 5 or more units of alcohol per week has been associated with a five times increased odds ratio of spontaneous abortions [29]. Similarly, moderate alcohol intake has been associated with an increased risk of stillbirth and infant mortality at a rate of 4.7 and 4.8 per 1,000 births, respectively [30]. It is possible that this unexpected mortality in the PAE+CHORIO cohort was associated with significantly altered maternal-placental-fetal hemodynamics in which the combination of insults exceeded a threshold of intrauterine inflammation secondary to oxidative stress, DNA damage, lipid peroxidation and trophoblast apoptosis and necrosis following PAE combined with up-regulation of cytokines and chemokines following both PAE and CHORIO [31–33]. We have previously shown that multiple cytokines including IL-1β, TNF-α and IL-6 are increased in rat offspring serum following CHORIO [20]. Chronic prenatal alcohol exposure has been observed to significantly increase levels of pro-inflammatory cytokines including intraleukin-6 (IL-6), IL-1β and tumor necrosis factor alpha (TNF-α) in both the human fetus and mother [34]. Future studies beyond the scope of the present investigation will elucidate the role of severe peripheral inflammation and postnatal survival, with a focus on pro-inflammatory cytokines in serum and placenta to further delineate the mechanisms of injury and identify a potential therapeutic target. Clinical studies have found a decrease in the frontal lobe volume following PAE, but the microstructural changes within this region have not been previously well described [35]. While it is understood that the frontal cortex is critical to executive functioning, the specific abnormalities within this region on MRI have not been characterized following PAE+CHORIO. The rodent medial frontal cortex contains networks that mediate higher-order behavior function and working memory [12]. Thus, in our PAE+CHORIO model, noting abnormalities in FA and RD on DTI within the medial frontal cortex reveals unique microstructural changes. Specifically, the significant increase in the RD within the medial frontal cortex of the PAE+CHORIO compared to controls and PAE alone most likely represents myelin damage within this region. Additionally, the AD and MD did not show any statistical differences in the PAE+CHORIO compared to PAE or controls alone in these frontal cortical regions, suggesting a diffuse difference in anisotropy as characterized by the decreased FA with no specific abnormalities in axonal health. While the direct impact on these changes to functional outcome remains to be described, future studies can investigate the correlation of these MRI findings with PAE induced changes in executive functioning and whether MRI changes could predict adverse neurobehavioral consequences in this patient population. Similarly, abnormalities within major white matter tracts on DTI as observed here, is consistent with the human studies on MRI changes in PAE [36,37]. While significance within the callosal white matter tract was not reached, the FA was decreasing in PAE+CHORIO compared to PAE and controls, indicating abnormalities in tissue coherence and microstructural organization in multiple brain regions. The FA within the capsular white matter tracts was noted to be significantly decreased, consistent with the vulnerability that white matter seems to have when exposed to these prenatal insults. Indeed, MRI shows microstructural brain abnormalities in children with PAE, including decreased FA in the splenium of the corpus callosum and reduced cortical volume, brain regions central to executive function [1,36,38–40].

## Limitations

One limitation of this study was the high rate of mortality observed in the PAE+CHORIO group, which resulted in a smaller number of offspring available for the studies. The mortality was unexpected and a clinically significant finding; the occurrence of PAE and CHORIO may occur in humans more frequently than previously thought, but the pregnancies may end in a spontaneous abortion before the cause is recognized due to the incompatibility with life. However, having the increased mortality also limits the preclinical investigations, thus resulting in smaller sample sizes in this study. Future studies should investigate potential mechanisms contributing to pregnancy failure after multiple in utero insults which could lead to a better understanding of the pathophysiology resultant in microstructural abnormalities on brain DTI and provide insights about the efficacy of therapeutic interventions in this vulnerable patient population.

## Conclusion

Further studies are ongoing to delineate the cellular and molecular mechanisms related to the severity of this injury and in utero demise, and brain injury in the surviving fetuses given our previous reports that inflammatory changes initiated in utero continue after birth [20]. The interaction of the maternal-placenta-fetal axis and the developing brain require additional investigations in order to identify the signaling mechanism through which this synergistic injury and in utero demise occurs. Strikingly, understanding of the detrimental changes that occur with this preclinical model will allow further investigation into potential therapeutic interventions, which could be used to aid the 5% of infants born in the United States exposed to alcohol prenatally and lessen the burden of the lifelong consequences.
